# Early post-transplantation factors predict survival outcomes in patients undergoing allogeneic hematopoietic cell transplantation for myelofibrosis

**DOI:** 10.1038/s41408-020-0302-9

**Published:** 2020-03-10

**Authors:** Tania Jain, Katie L. Kunze, Luke Mountjoy, Daniel K. Partain, Heidi Kosiorek, Nandita Khera, William J. Hogan, Vivek Roy, James L. Slack, Pierre Noel, Veena D. S. Fauble, Jose F. Leis, Lisa Sproat, Ayalew Tefferi, Mrinal M. Patnaik, Ruben A. Mesa, Jeanne Palmer

**Affiliations:** 1 Division of Hematology and Medical Oncology, Mayo Clinic, Phoenix, AZ USA; 2 Division of Biomedical Statistical and Informatics, Department of Health Sciences Research, Mayo Clinic, Phoenix, AZ USA; 3 Division of General Internal Medicine, Mayo Clinic, Rochester, MN USA; 4 Center for Palliative Medicine, Mayo Clinic, Rochester, MN USA; 5 Division of Hematology and Bone Marrow Transplant, Mayo Clinic, Rochester, MN USA; 6 Division of Hematology and Medical Oncology, Mayo Clinic, Jacksonville, FL USA; 7 Honor Health Cancer Care Network, Scottsdale, AZ USA; 8 University of Texas Health San Antonio Cancer Center, San Antonio, TX USA; 9 Present Address: Hematologic Malignancies and Bone Marrow Transplantation Program, Johns Hopkins University School of Medicine, Baltimore, MD USA

**Keywords:** Risk factors, Disease-free survival

## Abstract

Factors predicting allogeneic hematopoietic cell transplantation (HCT) outcomes in myelofibrosis in the early post-HCT period have not been defined thus far. We attempt to study such factors that can help identify patients at a higher risk of relapse or death. This retrospective study included 79 patients who underwent first HCT for myelofibrosis at three centers between 2005 and 2016. Univariate analysis showed that red blood cell (RBC) transfusion dependence (HR 9.02, 95% CI 4.0–20.35), platelet transfusion dependence (HR 8.17, 95%CI 3.83–17.37), 100% donor chimerism in CD33 + cells (HR 0.21, 95%CI 0.07–0.62), unfavorable molecular status (HR 4.41, 95%CI 1.87–10.39), normal spleen size (HR 0.42, 95%CI 0.19–0.94), grade ≥ 2 bone marrow fibrosis (vs. grade ≤ 1; HR 2.7, 95%CI 1.1–6.93) and poor graft function (HR 2.6, 95%CI 1.22–5.53) at day +100 were statistically significantly associated with relapse-free survival (RFS). RBC transfusion dependence and unfavorable molecular status were also statistically significant in the multivariate analysis. Patients in whom both of these factors were present had a significantly worse RFS when compared to those with one or none. While limited by a small sample size, we demonstrate the significance of transfusion dependence and molecular status at day +100 in predicting outcomes.

## Introduction

The therapeutic paradigm in myelofibrosis has evolved over the years, yet allogeneic hematopoietic cell transplantation (HCT) remains the only potentially curative treatment option. It is, however, fraught with risks of morbidities and mortality from the process of HCT itself and it remains an area of ongoing investigation how the outcomes of HCT in myelofibrosis could be further understood and improved. Various pre-HCT factors related to the patient, disease, and HCT itself have been associated with overall outcomes of HCT in myelofibrosis. Age and comorbidities at HCT have repeatedly been associated with HCT outcomes^1–3^. Pre-HCT dynamic international prognostic scoring system (DIPSS) and DIPSS Plus-based stratification has been shown to not only prognosticate outcomes in myelofibrosis overall, but also predict outcomes for patients undergoing HCT^4,5^. Somatic molecular mutations have also been described to have prognostic value in patients undergoing HCT for myelofibrosis with inferior outcomes associated with *U2AF1, ASXL1*, and *IDH2* mutations^6,7^. Similarly, donor source and intensity of conditioning regimen have been associated with outcomes in variable ways^2,8–11^.

Despite increased understanding of pre-HCT factors and improved outcomes from HCT overall, a considerable fraction of patients undergoing HCT for myelofibrosis either relapse and/or die of the disease. At this time, early post-HCT factors associated with a high risk of death from the disease or otherwise have not been described. Our study aimed to identify such factors at day +100 post-HCT, which are associated with long-term outcomes post-HCT. This can potentially help identify patients who may benefit from closer monitoring or an early intervention post-HCT.

## Materials and methods

### Patient selection

Patients who underwent a first HCT for myelofibrosis at one of the three sites of Mayo Clinic in Rochester MN, Scottsdale AZ, and Jacksonville FL between January 2005 and August 2016 were included in the study. Patients who died before day +100 were excluded from the analysis. We conducted a retrospective chart review to collect baseline patient, disease and HCT related data, day +100 characteristics, as well as information on outcomes post-HCT. Day +100 variables were collected if available between day +80 and day +120; and if more than one value was available, the one closest to day +100 was used. These included molecular mutation status, bone marrow fibrosis, spleen size, donor chimerism, presence of any grade of acute graft vs. host disease (GVHD), red blood cell (RBC), as well as platelet transfusion dependence, considering the various clinical features relevant to myelofibrosis and HCT.

### Definitions

RBC and platelet transfusion dependence was defined as transfusion requirement of over 2 units in a 4-week period of the respective products. The grade of bone marrow fibrosis was determined using World Health Organization criteria. High-risk cytogenetics were defined as presence of complex karyotype or single or two abnormalities involving +8, −7/−7q, i(17q), −5/−5q, inv(3), or 11q23 rearrangement as also referenced in DIPSS Plus score^12^. Since limited data was available on chimerism and driver mutation (*JAK2, CALR, or MPL*) status over the years, we used a variable called “*molecular status*” to reflect on molecular response after HCT. Molecular status was defined as “*favorable*” if either 100% donor chimerism in CD33 fraction from blood or absence of driver mutation was known. It was deemed “*unfavorable*” if donor chimerism was <100% or driver mutation was known to be positive at day +100. Relapse was defined as hematological (recurrence of cytopenias in the absence of graft failure), cytogenetic (reappearance of cytogenetic mutations) or molecular (reappearance of driver mutations or complete loss of donor chimerism). Poor graft function was defined as persistent cytopenias in at least 2 cell lines after day +100 with absolute neutrophil count <1500/mm^3^, platelet count <30/μL, hemoglobin <8.5 g/dL. Primary graft failure was defined as <5% donor chimerism at any time after HCT, or infusion of donor lymphocyte infusion due to permanent loss of neutrophils or platelets, or <50% donor chimerism despite treatment with donor lymphocyte infusion.

### Statistics

Descriptive statistics were estimated, and Chi-squared tests and analysis of variance (ANOVA) were used to examine group differences. Variables of interest included patient characteristics, disease characteristics, and treatment regimens. Univariate Cox proportional hazard models for pre-HCT and post-HCT variables of interest were used to examine differences in relapse-free survival (RFS) and overall survival (OS). Multivariate Cox proportional hazard models were estimated using statistically significant variables and variables of importance (associations with a *p*-value of <0.1 in the univariate models) and taking into account the number of events per variable to predict RFS and OS. Additional analyses explored relationships between RBC transfusion dependence and ABO incompatibility at the time of HCT, as well as, between graft failure and poor graft function and OS using Chi-squared tests. Kaplan–Meier (K–M) curves of RBC transfusion dependence and molecular status at day +100 predicting RFS were estimated. Finally, differences in survival for the number of risk factors were examined by calculating how many risk factors (none, some combination of included factors, or all factors in the multivariate analysis) each patient had for the variables included in the multivariate analysis and a K–M curve predicting RFS was estimated.

## Results

### Baseline patient data

A total 87 patients underwent HCT for myelofibrosis during the years 2005 and 2016 at the three centers. Of these, eight died within the first 100 post-HCT and were not included in the analysis. Among the 79 patients included in the analysis, median age at the time of HCT was 58 (range, 19–73) years and 59% were men. The baseline information are tabulated in Table 1. *JAK2* mutation status was available for 76 out of 79 patients, and was positive in 41 (54%) of these 76 patients. *CALR* and *MPL* mutation testing was only started in recent years since the description^13–15^, it was not available in many patients. Since *JAK2, CALR*, and *MPL* mutations are known to be mutually exclusive, *CALR* and *MPL* mutations testing was only explored for patients who were *JAK2* mutation negative. Of these, *CALR* mutation status was available in 21 patients and was known to be positive in 8 (38%) while *MPL* mutation was available in 31 and positive in 4 (13%) patients. Twenty-four (31%) patients were treated with *JAK* inhibitor therapy prior to HCT while 10 (13%) had undergone splenectomy prior to HCT. Additional baseline and HCT related information is tabulated in Table 1. Donor source was matched related in 36 (46%) patients while matched unrelated in 34 (43%), mismatched related (at single allele) in 1 (1%), single-allele mismatched unrelated in 6 (8%) and haploidentical relative in 2 (3%). ABO compatibility between the donor and recipient was assessed using standard criteria to further elaborate on RBC transfusion dependence as explained below^16^. Anti-thymocyte globulin (ATG) was used as a part of the GVHD prophylaxis in 42% patients and has been the treating physician’s discretion at our institution.

**Table 1:** Baseline patient, disease, and transplantation characteristics.

Characteristics	*N*, total 79 (%)
Median age at diagnosis in years (range)	56 (19–73)
Median age at HCT in years (range)	58 (19–73)
Female gender	32 (41)
Myelofibrosis type	
Primary	54 (68)
Post-ET/Post-PV	25 (32)
DIPSS category	
Low/intermediate-1	4 (5)
Intermediate-2	71 (90)
High	4 (5)
RBC transfusion dependence at HCT	54 (68)
Platelet transfusion dependence at HCT	10 (13)
High-risk cytogenetics at SCT (*n* = 68)	16 (24)
JAK2 mutation (*n* = 76)	
Positive	41 (52)
Negative	35 (44)
JAK inhibitor treatment prior to HCT	24 (31)
Conditioning regimen:	
Myeloablative	14 (18)
Reduced intensity	65 (82)
Anti-thymocyte globulin administered	33 (42)
Donor source:	
Matched related	36 (46)
Others	43 (54)
Graft type:	
Peripheral blood	77 (97)
Bone marrow	2 (3)
Graft vs. host disease prophylaxis:	
Calcineurin inhibitor + Methotrexate	56 (71)
Calcineurin inhibitor + Mycophenolate	20 (25)
Other	3 (4)
ABO incompatibility (*n* = 75):	
None (Compatible)	43 (57)
Minor	19 (25)
Major	12 (16)
Bidirectional	1 (1)

*DIPSS* Dynamic International Prognostic Scoring System, *ET* essential thrombocythemia, *HCT* allogeneic hematopoietic cell transplantation, *PV* polycythemia vera, *RBC* red blood cells.

### Day +100 outcomes

At day +100, 27 out of 79 (34%) were RBC transfusion dependent and 21 (27%) were platelet transfusion dependent. Donor chimerism was 100% in CD3 + cells in 33 (42%), 100% in CD33 + cells in 48 (61%) patients and was not available in 23 patients. Along with information on driver mutations as available, we were able to determine molecular status on 72 patients, of which 60 (83%) were favorable and 12 (17%) were unfavorable.

Spleen size was available both at the time of HCT and at day +100 in 59 patients out of the 69 patients who did not have a prior splenectomy. Of note, two additional patients out of the 69, underwent splenectomy after HCT by day +100. Of these 59 patients, spleen size had decreased in 48 (81%), increased in one (by 1 cm) and unchanged in 10 patients (17%, of which 6 were normal size at the time of HCT). Median decrease in spleen size in the aforementioned 48 patients was 4.8 (range, 1–10.6) cm by day +100.

Bone marrow fibrosis grade at the time of HCT was grade 1 in 5 (7%), grade 2 in 16 (21%), grade 3 in 55 (72%) out of the 76 available bone marrow fibrosis information. No one had grade 0 fibrosis. However, by day +100, 9 (13%) patients had grade 0 fibrosis, 20 (28%) had grade 1 fibrosis, 22 (31%) had grade 2 fibrosis while 21 (29%) had grade 3 fibrosis. The grade of bone marrow fibrosis was available at both pre-HCT and at day +100, in 69 out of 79 patients. Of these, two (3%) had worsening fibrosis compared to pre-HCT marrow assessment—one had overt disease progression at 7 months post-HCT and died subsequently while the other is alive without disease relapse/progression at 30 months at last follow-up. Forty (58%) patients were noted to have an improvement in bone marrow fibrosis by at least one grade while it was unchanged in 27 (39%) patients.

### Overall HCT outcomes

The median follow-up on the study was 31 (range, 3.1–119.2) months. Twenty-six (33%) patients died and 15 (19%) relapsed at the time of this study. Cause of death was relapse or disease progression in 8/26 (31%), GVHD in 8/26 (31%), infection in 6/26 (23%) and other transplant related causes in 4/26 (15%) patients. An additional 7 (9%) patients relapsed but were alive at the time of the last follow-up. A comparison of various patient and disease characteristics in patients who had a disease relapse vs. those who did not, is shown in Supplementary Table 1. Primary graft failure was noted in one patient while poor graft function was noted in 19 (24%) patients.

### Univariate and multivariate analysis

The univariate Cox Proportional Hazards model was conducted to identify day +100 factors that were significantly associated with RFS and OS. Since, pre-HCT factors are also known to affect day +100, as well as overall outcomes of HCT, they were also included in these models to identify any potential confounding factors.

Univariate analyses for RFS showed that among pre-HCT factors, RBC transfusion dependence prior to HCT (hazard ratio (HR) 2.87, 95% confidence interval (CI) 1.09–7.55, *p* = 0.032) and high-risk cytogenetics (HR 2.54, 95%CI 1.10–5.87, *p* = 0.029) were statistically significantly associated. Among day +100 post-HCT factors, RBC transfusion dependence (HR 9.02, 95%CI 4.0–20.35, *p* < 0.001), platelet transfusion dependence (HR 8.17, 95% CI 3.83–17.37, *p* < 0.001), 100% donor chimerism in CD33 + cells (HR 0.21, 95% CI 0.07–0.62, *p* = 0.004), unfavorable molecular status (HR 4.41, 95% CI 1.87–10.39, *p* < 0.001), normal spleen size at day +100 (HR 0.42, 95% CI 0.19–0.94, *p* = 0.035), grade ≥ 2 of bone marrow fibrosis (vs. grade ≤ 1; HR 2.7, 95% CI 1.1–6.93, *p* = 0.031) and poor graft function (HR 2.6, 95% CI 1.22–5.53, *p* = 0.013) were statistically significantly associated with RFS. The complete analysis is shown in Table 2.

**Table 2:** Univariate Cox Proportional Hazards models of pre- and post-transplantation factors predicting relapse-free survival.

	Hazard ratio (95% CI for HR)	*p*-value
Pre-transplantation factors		
JAK2 mutation present	1.02 (0.48–2.16)	0.96
Age at transplant (>55 years vs. <55 years)	1.39 (0.61–3.12)	0.43
DIPSS stratification (high risk vs. intermediate-2)	1.76 (0.41–7.45)	0.45
JAK inhibitor therapy prior to SCT	0.93 (0.41–2.11)	0.86
Pre-HCT RBC transfusion dependence	2.87 (1.09–7.55)	**0.03**
Pre-HCT platelet transfusion dependence	0.65 (0.19–2.16)	0.49
High risk cytogenetics	2.54 (1.10–5.87)	**0.03**
Conditioning regimen (reduced intensity vs. myeloablative)	0.74 (0.30–1.83)	0.52
Graft vs. host disease prophylaxis (Calcineurin inhibitor + methotrexate vs. Calcineurin inhibitor + mycophenolate)	0.98 (0.43–2.24)	0.97
Donor source (matched related vs. other)	0.57 (0.27–1.21)	0.15
Anti-thymocyte globulin administered	0.63 (0.29–1.37)	0.25
Post-transplantation (day +100) Factors		
RBC transfusion dependence	9.02 (4.00–20.35)	**<0.001**
Platelet transfusion dependence	8.16 (3.84–17.37)	**<0.001**
100% Donor chimerism in CD3 + cells	1.01 (0.37–2.79)	0.98
100% Donor chimerism in CD33 + cells	0.21 (0.07–0.62)	**0.004**
Grade ≥2 bone marrow fibrosis (vs. Grade ≤1)	2.76 (1.10–6.93)	**0.03**
No improvement in bone marrow fibrosis	1.52 (0.69–3.33)	0.30
Acute graft vs. host disease (grades 2–4 vs. Grade 0–1)	1.04 (0.32–3.44)	0.95
Normal spleen size	0.42 (0.19–0.94)	**0.04**
Unfavorable molecular status	4.4 (4.87–10.39)	**<0.001**
Poor graft function day +100	2.60 (1.22–5.53)	0.01

*DIPSS* Dynamic International Prognostic Scoring System, *HCT* Allogeneic hematopoietic cell transplantation, *RBC* red blood cells.

The statistically significant values are in bold.

**Fig. 1:**
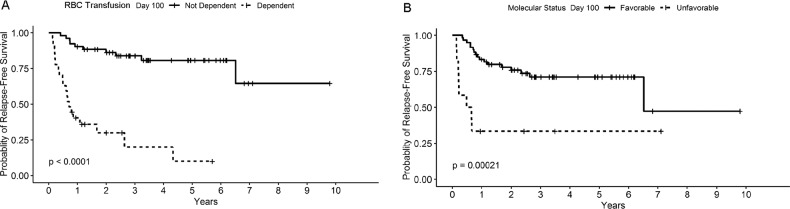
Kaplan–Meier curves for RBC transfusion dependence and molecular status association with relapse-free survival. **a** RBC transfusion dependence, **b** Molecular status.

**Fig. 2:**
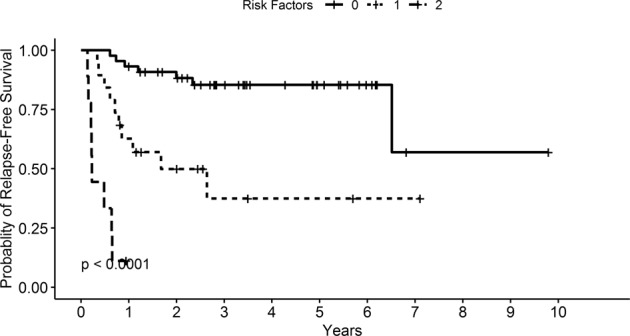
Probability of relapse-free survival by presence of number of risk factors.

For the multivariate analysis, in line with the recommended ratio of ≥5–9 events per variable, two variables were included as predictors^17^. Owing to various missing results, donor chimerism in CD33 + cells at day +100 could not be included in the multivariate model. Additionally, RBC transfusion dependence at HCT was not significantly different from RBC transfusion dependence at day +100 when analyzed separately (*χ*^2^ = 0.28, *p* = 0.59). Therefore, only RBC transfusion dependence at day +100 was included in the multivariate analysis. Finally, due to a statistically significant association between RBC transfusion dependence and platelet transfusion dependence (*r* 0.77, 95% CI 0.67–0.85, *p* < 0.001), it was deemed appropriate to only include one out of these; and hence, only RBC transfusion dependence at day +100 was added to the multivariate model as it showed a larger HR in the univariate model. After consideration of these factors and the number of events (deaths and/or relapses), RBC dependence and molecular status at day +100 were included in the multivariate analysis. Both RBC dependence (HR 11.4, 95% CI 4.28–30.51, *p* < 0.001) and unfavorable molecular status (HR 3.18, 95% CI 1.30–7.82, *p* = 0.012) were statistically significant in this multivariate model as shown in Table 3. Kaplan–Meier survival curves for these factors are shown in Fig. 1. An additional analysis examined differences in RFS for those who had none, one, or both of these risk factors and showed a significant difference in RFS among these patients (Fig. 2).

**Table 3:** Multivariate analysis for factors predicting relapse-free survival.

	HR	CI.lower.HR	CI.upper.HR	*p*-value
RBC transfusion dependence day +100	11.43	4.28	30.51	**<0.001**
Unfavorable molecular status day +100	3.18	1.30	7.82	**0.01**

CI confidence interval, RBC red blood cells.

The statistically significant values are in bold.

Similar univariate and multivariate models were conducted for OS as endpoint. Among the day +100 factors studied (Table 4), RBC transfusion dependence (HR 8.1, 95% CI 3.5–18.8, *p* < 0.001), platelet transfusion dependence (HR 7.2, 95% CI 3.3–15.9, *p* < 0.001), donor chimerism in CD33 + cells (HR 0.3, 95% CI 0.9–0.91, *p* = 0.03), and unfavorable molecular status (HR 4.10, 95% CI 1.64–10.24, *p* = 0.003) were statistically significantly associated with OS in these patients. On multivariate analysis, RBC transfusion dependence at day +100 was significantly associated with OS (HR = 9.2, 95% CI 3.2–26.3, *p* = <0.001) while molecular status at day +100 showed a possible association not deemed statistically significant (HR = 2.23, 95% CI 0.86–5.76, *p* = 0.099) as shown in Table 5. The Kaplan–Meier curve for RBC transfusion dependence is illustrated in Supplementary Fig. 1. It is, however, important to note that the hazard ratio for RBC transfusion dependence is high and has an even wider confidence interval, which is likely a result of small sample size.

**Table 4:** Univariate Cox Proportional Hazards models of pre- and post-transplantation factors predicting overall survival.

	Hazard ratio (95% CI for HR)	*p*-value
Pre-transplantation factors		
JAK2 mutation present	0.76 (0.34–1.70)	0.51
Age at transplant (>55 years vs. <55 years)	1.18 (0.51–2.72)	0.69
DIPSS stratification (high risk vs. intermediate-2)	2.21 (0.52–9.47)	0.28
JAK inhibitor therapy prior to SCT	0.93 (0.38–2.23)	0.86
Pre-HCT RBC transfusion dependence	4.36 (1.31–14.60)	**0.02**
Pre-HCT platelet transfusion dependence	0.76 (0.23–2.52)	0.65
High risk cytogenetics	3.04 (1.26–7.34)	**0.01**
Conditioning regimen (reduced intensity vs. myeloablative)	0.59 (0.24–1.49)	0.27
Graft vs. host disease prophylaxis (Calcineurin inhibitor + methotrexate vs. Calcineurin inhibitor + mycophenolate)	0.79 (0.32–2.02)	0.64
Donor source (matched related vs. other)	0.66 (0.30–1.45)	0.30
Anti-thymocyte globulin administered	0.45 (0.19–1.07)	0.07
Post-transplantation (day +100) factors		
RBC transfusion dependence	8.06 (3.45–18.85)	**<0.001**
Platelet transfusion dependence	7.24 (3.29–15.95)	**<0.001**
100% Donor chimerism in CD3 + cells	1.01 (0.33–3.08)	0.99
100% Donor chimerism in CD33 + cells	0.28 (0.09–0.91)	**0.03**
Grade ≥2 bone marrow fibrosis (vs. Grade ≤1)	2.72 (1.00–7.41)	**0.05**
No improvement in bone marrow fibrosis	1.96 (0.84–4.55)	0.12
Acute GVHD (Grades 2–4 vs. Grade 0–1)	0.96 (0.29–3.20)	0.95
Normal spleen size	0.55 (0.24–1.29)	0.17
Unfavorable molecular status	4.1 (1.64–10.24)	**0.003**
Poor graft function day +100	2.06 (0.92–4.64)	0.08

*DIPSS* Dynamic International Prognostic Scoring System, *HCT* allogeneic hematopoietic cell transplantation, *RBC* red blood corpuscles.

The statistically significant values are in bold.

**Table 5:** Multivariate analysis for factors predicting overall survival.

	HR	CI.lower.HR	CI.upper.HR	*p*-value
RBC transfusion dependence day +100	9.24	3.24	26.33	**<0.001**
Unfavorable molecular status day +100	2.23	0.86	5.76	0.099

*CI* confidence interval, *RB*C red blood cells.

The statistically significant values are in bold.

### Additional analysis

Since ABO incompatibility is known to result in RBC transfusion dependence, we further studied if the RBC transfusion dependence at day +100 was a possible result of ABO incompatibility at HCT. These results are shown in Supplementary Table 2. A chi-square test was performed to study this association and did not show a significant difference in rates of ABO types for those who did and did not experiences RBC transfusion dependence in this patient population.

## Discussion

HCT is known to be the only potentially curative treatment for myelofibrosis but a high risk of mortality has been associated with the treatment. Our study provides a potential strategy to identify patients at high risk of a poor outcome following HCT at an early time point of day +100. This would lead the way to study if these patients would benefit from interventions such as closer monitoring of clinical status, early withdrawal of immunosuppression (when feasible) or pre-emptive therapy such as maintenance therapy or donor lymphocyte infusion. The utility of these interventions, however, remains to be studied. Transfusion dependence at day +100 appeared to be most strongly associated with inferior RFS, as well as OS as shown in both the univariate, as well as multivariate analysis in our study. This was shown to be independent of ABO incompatibility between the donor and the recipient. Owing to limited number of patients, we were not able to study the reasons for transfusion dependence in a statistical model; and was most commonly attributed to slow engraftment post-HCT.

We use a combined variable for assessment of impact of molecular status as both donor chimerism and the mutation status have been identified as indicators of measurable residual disease and since both detect persistence of recipient DNA^18^. We found that patients with a favorable molecular status, either by full donor chimerism or by absence of driver mutation by day +100, are more likely to have better RFS. Previously clearance of *JAK2 v617F* mutation has been shown to correlate with lower risk of relapse in these patients^19^. Similarly, MPL mutation clearance post-HCT has been associated with hematological recovery and correlate with donor chimerism^20^. These along with our data indicate that driver mutation status by itself, or combined with chimerism status can shed important prognostic information about these patients in the early post-HCT period to allow consideration of possible interventions. Pre-emptive donor lymphocyte infusion has been explored in patients with myelofibrosis with a higher success noted in patients who were treated with molecular relapse (100%) than those with overt relapse (44%), again suggesting the importance of an early or pre-emptive intervention^21^. These patients could be potential candidates of interest for studies using maintenance/ pre-emptive intervention following HCT in myelofibrosis.

Bone marrow fibrosis was noted to improve in around 60% patients by day +100 although the improvement in fibrosis did not seem to be correlated with RFS. A higher grade of fibrosis at day +100, however, did correlate with inferior RFS in the univariate analysis. A similar finding has been demonstrated by Kroger et al.^22^ where bone marrow fibrosis grade 0–1 at day +100 was associated with improved survival at 5 years. More recently, therapies such as PRM-151 have shown the potential to improve bone marrow fibrosis^23^. Tumor necrosis growth factor beta has also shown to be a major contributor to the pathogenesis of the fibrosis and therapies targeting these are being developed^24–27^. Whether the use of such strategies in the post-HCT period will result in improved outcomes warrants additional studies.

Our study, while studying predictive factors at day +100 for the first time in myelofibrosis, is limited by its retrospective nature and limited sample size. Owing to a smaller sample in the multivariate model, the hazard ratios showed high values and confidence intervals showed wide ranges. Also, since the first full assessment of the marrow is usually done at day +100, we could not include patients who died prior to day +100 in this analysis, which results in selection bias, eliminating patients who died early within 100 days from HCT. Given the limited scope of this study, larger or possibly registry data can be studied to further investigate these findings.

## Conclusion

In this exploratory analysis of day +100 variables for predicting long-term outcomes in patients undergoing HCT for MF, we report a strong relationship between transfusion dependence of any etiology at day +100 and increased risk of relapse/death. These patients should, perhaps, be considered for a closer monitoring or an early intervention. Larger studies will be needed to confirm these findings before any management recommendations can be suggested.

## Supplementary information

Supplementary material

## References

[1] Robin M (2019). Long-term outcome after allogeneic hematopoietic cell transplantation for myelofibrosis. Haematologica.

[2] Kroger N (2009). Allogeneic stem cell transplantation after reduced-intensity conditioning in patients with myelofibrosis: a prospective, multicenter study of the Chronic Leukemia Working Party of the European Group for Blood and Marrow Transplantation. Blood.

[3] Sorror ML (2005). Hematopoietic cell transplantation (HCT)-specific comorbidity index: a new tool for risk assessment before allogeneic HCT. Blood.

[4] Scott BL (2012). The dynamic International Prognostic Scoring System for myelofibrosis predicts outcomes after hematopoietic cell transplantation. Blood.

[5] Ditschkowski M (2012). Dynamic International Prognostic Scoring System scores, pre-transplant therapy and chronic graft-versus-host disease determine outcome after allogeneic hematopoietic stem cell transplantation for myelofibrosis. Haematologica.

[6] Tamari R (2019). Impact of high-molecular-risk mutations on transplantation outcomes in patients with myelofibrosis. Biol. Blood Marrow Transplant..

[7] Kroger N (2017). Impact of molecular genetics on outcome in myelofibrosis patients after allogeneic stem cell transplantation. Biol. Blood Marrow Transplant..

[8] Gupta V (2014). Reduced-intensity hematopoietic cell transplantation for patients with primary myelofibrosis: a cohort analysis from the center for international blood and marrow transplant research. Biol. Blood Marrow Transplant..

[9] Rondelli D (2014). MPD-RC 101 prospective study of reduced-intensity allogeneic hematopoietic stem cell transplantation in patients with myelofibrosis. Blood.

[10] Jain T (2019). Comparison of reduced intensity conditioning regimens used in patients undergoing hematopoietic stem cell transplantation for myelofibrosis. Bone Marrow Transplant.

[11] Robin M (2016). Outcome after transplantation according to reduced-intensity conditioning regimen in patients undergoing transplantation for myelofibrosis. Biol. Blood Marrow Transplant..

[12] Gangat N (2011). DIPSS plus: a refined dynamic international prognostic scoring system for primary myelofibrosis that incorporates prognostic information from karyotype, platelet count, and transfusion status. J. Clin. Oncol..

[13] Klampfl T (2013). Somatic mutations of calreticulin in myeloproliferative neoplasms. N. Engl. J. Med..

[14] Nangalia J (2013). Somatic CALR mutations in myeloproliferative neoplasms with nonmutated JAK2. N. Engl. J. Med..

[15] Pikman Y (2006). MPLW515L is a novel somatic activating mutation in myelofibrosis with myeloid metaplasia. PLoS Med..

[16] Booth GS, Gehrie EA, Bolan CD, Savani BN (2013). Clinical guide to ABO-incompatible allogeneic stem cell transplantation. Biol. Blood Marrow Transplant..

[17] Vittinghoff E, McCulloch CE (2007). Relaxing the rule of ten events per variable in logistic and Cox regression. Am. J. Epidemiol..

[18] Srour, S. A. et al. Mixed myeloid chimerism and relapse of myelofibrosis after allogeneic stem cell transplantation. *Haematologica* (2019). [Epub ahead of print]. 10.3324/haematol.2019.223503 PMC8252931 31296578

[19] Alchalby H (2010). Impact of JAK2V617F mutation status, allele burden, and clearance after allogeneic stem cell transplantation for myelofibrosis. Blood.

[20] Alchalby H (2010). Screening and monitoring of MPL W515L mutation with real-time PCR in patients with myelofibrosis undergoing allogeneic-SCT. Bone Marrow Transplant..

[21] Kroger N (2009). JAK2-V617F-triggered preemptive and salvage adoptive immunotherapy with donor-lymphocyte infusion in patients with myelofibrosis after allogeneic stem cell transplantation. Blood.

[22] Kroger N (2014). Dynamic of bone marrow fibrosis regression predicts survival after allogeneic stem cell transplantation for myelofibrosis. Biol. Blood Marrow Transplant..

[23] Verstovsek S (2015). PRM-151 in myelofibrosis: durable efficacy and safety at 72 weeks. Blood.

[24] Wang JC (2006). Quantitative analysis of growth factor production in the mechanism of fibrosis in agnogenic myeloid metaplasia. Exp. Hematol..

[25] Vannucchi AM (2005). A pathobiologic pathway linking thrombopoietin, GATA-1, and TGF-beta1 in the development of myelofibrosis. Blood.

[26] Schmitt A (2002). Polymorphonuclear neutrophil and megakaryocyte mutual involvement in myelofibrosis pathogenesis. Leuk. Lymphoma.

[27] Mascarenhas J (2014). Anti-transforming growth factor-beta therapy in patients with myelofibrosis. Leuk. Lymphoma.

