# Supplementary material for: Early post-transplantation factors predict survival outcomes in patients undergoing allogeneic hematopoietic cell transplantation for myelofibrosis
Source: Blood Cancer J. 2020 Mar 10;10(3):36. doi: 10.1038/s41408-020-0302-9 (PMC7064504; doi:10.1038/s41408-020-0302-9)
Supplement: Supplementary file 1 — Supplementary material [file 41408_2020_302_MOESM1_ESM.docx]

**Supplementary Legend**

Supplementary Table 1: Patient characteristics by occurrence of relapse

Supplementary Table 2: Analysis of ABO incompatibility and RBC transfusion dependence at day +100

**Supplementary Figure 1: Kaplan Meier curves for RBC transfusion dependence with overall survival**

**Supplementary Table 1: Patient characteristics by occurrence of relapse**

|  | No Relapse (N=64) | Relapse (N=15) | Total (N=79) | p value |
| --- | --- | --- | --- | --- |
| **Age at Transplant (in years)** |  |  |  | 0.412^1^ |
| Mean (SD) | 56.594 (9.682) | 58.800 (7.466) | 57.013 (9.299) |  |
| Median | 58.000 | 60.000 | 58.000 |  |
| Range | 19.000 - 73.000 | 42.000 - 69.000 | 19.000 - 73.000 |  |
| **Age at Diagnosis (in years)** |  |  |  | 0.724^1^ |
| Mean (SD) | 54.469 (9.592) | 55.467 (10.690) | 54.658 (9.745) |  |
| Median | 56.000 | 57.000 | 56.000 |  |
| Range | 19.000 - 73.000 | 32.000 - 67.000 | 19.000 - 73.000 |  |
| **Gender** |  |  |  | 0.589^2^ |
| Male | 39 (60.9%) | 8 (53.3%) | 47 (59.5%) |  |
| Female | 25 (39.1%) | 7 (46.7%) | 32 (40.5%) |  |
| **Diagnosis at HCT** |  |  |  | 0.165^2^ |
| Primary MF | 46 (71.9%) | 8 (53.3%) | 54 (68.4%) |  |
| Secondary MF | 18 (28.1%) | 7 (46.7%) | 25 (31.6%) |  |
| **DIPSS Risk Category** |  |  |  | 0.352^2^ |
| Int-1 | 4 (6.2%) | 0 (0.0%) | 4 (5.1%) |  |
| Int-2 | 56 (87.5%) | 15 (100.0%) | 71 (89.9%) |  |
| High | 4 (6.2%) | 0 (0.0%) | 4 (5.1%) |  |
| **RBC Transfusion Dependency (pre-HCT)** |  |  |  | 0.281^2^ |
| Not Dependent | 22 (34.4%) | 3 (20.0%) | 25 (31.6%) |  |
| Dependent | 42 (65.6%) | 12 (80.0%) | 54 (68.4%) |  |
| **Platelet Transfusion Dependency (pre-HCT)** |  |  |  | 0.342^2^ |
| Not Dependent | 57 (89.1%) | 12 (80.0%) | 69 (87.3%) |  |
| Dependent | 7 (10.9%) | 3 (20.0%) | 10 (12.7%) |  |
| **High risk cytogenetics at HCT** |  |  |  | 0.032^2^ |
| Not High Risk | 45 (81.8%) | 7 (53.8%) | 52 (76.5%) |  |
| High Risk | 10 (18.2%) | 6 (46.2%) | 16 (23.5%) |  |
| **Molecular Status (Day 100, favorable/unfavorable)** |  |  |  | < 0.001^2^ |
| Favorable | 54 (90.0%) | 6 (50.0%) | 60 (83.3%) |  |
| Unfavorable | 6 (10.0%) | 6 (50.0%) | 12 (16.7%) |  |
| **JAK positive molecular status at HCT/diagnosis** |  |  |  | 0.693^2^ |
| Positive | 33 (51.6%) | 8 (53.3%) | 41 (51.9%) |  |
| Negative | 28 (43.8%) | 7 (46.7%) | 35 (44.3%) |  |
| **JAK inhibitor prior to HCT** |  |  |  | 0.811^2^ |
| No | 44 (69.8%) | 10 (66.7%) | 54 (69.2%) |  |
| Yes | 19 (30.2%) | 5 (33.3%) | 24 (30.8%) |  |
| **Conditioning Regimen** |  |  |  | 0.797^2^ |
| Myeloablative | 11 (17.2%) | 3 (20.0%) | 14 (17.7%) |  |
| Reduced intensity | 53 (82.8%) | 12 (80.0%) | 65 (82.3%) |  |
| **Donor Source** |  |  |  | 0.630^2^ |
| Other Types | 34 (53.1%) | 9 (60.0%) | 43 (54.4%) |  |
| Matched/Related | 30 (46.9%) | 6 (40.0%) | 36 (45.6%) |  |
| **Graft type** |  |  |  | 0.257^2^ |
| Peripheral Blood | 63 (98.4%) | 14 (93.3%) | 77 (97.5%) |  |
| Bone Marrow | 1 (1.6%) | 1 (6.7%) | 2 (2.5%) |  |
| **GVHD prophylaxis** |  |  |  | 0.096^2^ |
| CNI + Methotrexate | 46 (71.9%) | 10 (66.7%) | 56 (70.9%) |  |
| CNI + Mycophenolate | 17 (26.6%) | 3 (20.0%) | 20 (25.3%) |  |
| Other | 1 (1.6%) | 2 (13.3%) | 3 (3.8%) |  |
| **ABO Incompatibility (recoded)** |  |  |  | 0.738^2^ |
| Compatible/Minor | 50 (82.0%) | 12 (85.7%) | 62 (82.7%) |  |
| Major/Bidirectional | 11 (18.0%) | 2 (14.3%) | 13 (17.3%) |  |
| **ATG Administered** |  |  |  | 0.840^2^ |
| No | 36 (57.1%) | 9 (60.0%) | 45 (57.7%) |  |
| Yes | 27 (42.9%) | 6 (40.0%) | 33 (42.3%) |  |
| **CALR Mutation** |  |  |  | 0.586^2^ |
| Yes | 6 (35.3%) | 2 (50.0%) | 8 (38.1%) |  |
| No | 11 (64.7%) | 2 (50.0%) | 13 (61.9%) |  |
| **ABO Incompatibility** |  |  |  | 0.257^2^ |
| Compatible | 37 (61.7%) | 6 (42.9%) | 43 (58.1%) |  |
| Minor | 13 (21.7%) | 6 (42.9%) | 19 (25.7%) |  |
| Major | 10 (16.7%) | 2 (14.3%) | 12 (16.2%) |  |
| **GVHD Grade** |  |  |  | 0.793^2^ |
| Grade 0-1 | 7 (10.9%) | 2 (13.3%) | 9 (11.4%) |  |
| Grades 2-4 | 57 (89.1%) | 13 (86.7%) | 70 (88.6%) |  |
| **Grade of BM Fibrosis (pre-HCT)** |  |  |  | 0.834^2^ |
| Grade 1 | 4 (6.6%) | 1 (6.7%) | 5 (6.6%) |  |
| Grade 2 | 12 (19.7%) | 4 (26.7%) | 16 (21.1%) |  |
| Grade 3 | 45 (73.8%) | 10 (66.7%) | 55 (72.4%) |  |
| **Spleen Size (Day 100, Normal vs. Abnormal)** |  |  |  | 0.018^2^ |
| Abnormal | 24 (40.7%) | 10 (76.9%) | 34 (47.2%) |  |
| Normal | 35 (59.3%) | 3 (23.1%) | 38 (52.8%) |  |
| **BM Fibrosis (Day 100)** |  |  |  | 0.010^2^ |
| Grade 0/1 | 27 (46.6%) | 1 (7.7%) | 28 (39.4%) |  |
| Grade 2/3 | 31 (53.4%) | 12 (92.3%) | 43 (60.6%) |  |
| **RBC Transfusion Dependence (Day 100)** |  |  |  | < 0.001^2^ |
| Not Dependent | 49 (76.6%) | 3 (20.0%) | 52 (65.8%) |  |
| Dependent | 15 (23.4%) | 12 (80.0%) | 27 (34.2%) |  |
| **Platelet Transfusion Dependence (Day 100)** |  |  |  | 0.001^2^ |
| Not Dependent | 52 (81.2%) | 6 (40.0%) | 58 (73.4%) |  |
| Dependent | 12 (18.8%) | 9 (60.0%) | 21 (26.6%) |  |
| **CD3D100** |  |  |  | 0.579^2^ |
| less than 100% donor | 19 (39.6%) | 4 (50.0%) | 23 (41.1%) |  |
| 100% donor | 29 (60.4%) | 4 (50.0%) | 33 (58.9%) |  |
| **CD33D100** |  |  |  | 0.005^2^ |
| less than 100% donor | 4 (8.5%) | 4 (44.4%) | 8 (14.3%) |  |
| 100% donor | 43 (91.5%) | 5 (55.6%) | 48 (85.7%) |  |

1. Linear Model ANOVA
2. Pearson’s Chi-squared test

*percentages adjusted for missing values

**Supplementary Table 2: Analysis of ABO incompatibility and RBC transfusion dependence at day +100**

|  | Not RBC Transfusion Dependent, N=52 (%) | RBC Transfusion Dependent, N=27(%) | p value |
| --- | --- | --- | --- |
| **ABO Incompatibility** |  |  | 0.095 |
| Compatible | 31 (62%) | 12 (48%) |  |
| Minor | 14 (28%) | 5 (20%) |  |
| Major | 5 (10%) | 7 (28%) |  |
| Bidirectional | 0 | 1 (4%) |  |

**Supplementary Figure 1: Kaplan Meier curves for RBC transfusion dependence with overall survival**

**
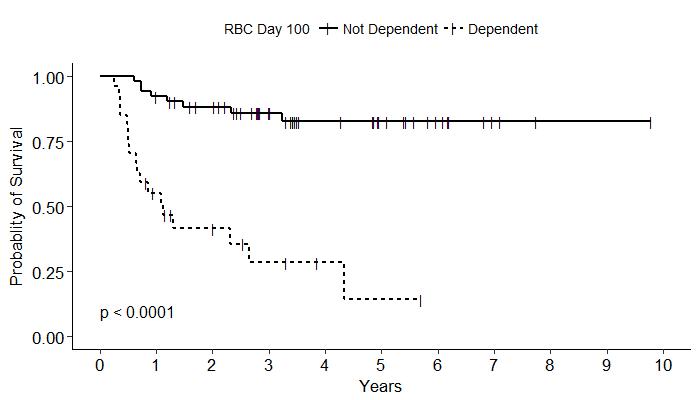
**
